# Hypoxia-Driven Functional Conversion of CAPE: From Anti-Inflammatory to Pro-Tumorigenic Action in the Human Astrocytoma Cell Line CCF-SSTG1

**DOI:** 10.3390/molecules31010140

**Published:** 2025-12-31

**Authors:** Anna Kurek-Górecka, Małgorzata Kłósek, Grażyna Pietsz, Radosław Balwierz, Zenon P. Czuba

**Affiliations:** 1 Department of Microbiology and Immunology, Medical University of Silesia in Katowice, Jordana 19, 41-808 Zabrze, Poland; 2 Institute of Chemistry, University of Opole, Oleska 48, 45-052 Opole, Poland

**Keywords:** CAPE, metalloproteinase, cytokines, glioblastoma, anti-inflammatory, chemoprevention

## Abstract

The glioblastoma multiforme (GBM) microenvironment, characterized by hypoxia and inflammation, is a principal driver of therapeutic resistance. Although natural compounds such as Caffeic Acid Phenethyl Ester (CAPE) are investigated for their anti-neoplastic properties, their bioactivity within the distinct metabolic landscape of the tumor core remains to be fully elucidated. Taking advantage of the recognized immunomodulatory properties of CAPE and its ability to cross the blood–brain barrier, we hypothesized that hypoxia is a key factor determining its effect on glioma-associated inflammation. To test this hypothesis, we investigated the immunomodulatory effects of CAPE on the human astrocytoma cell line CCF-STTG1. Cells were cultured under normoxic and hypoxic conditions, stimulated with lipopolysaccharide (LPS) and interferon-alpha (IFN-α) to induce an inflammatory phenotype, and subsequently treated with CAPE. The secretion profiles of key cytokines (IL-8, IL-10, IL-26) and matrix metalloproteinases (MMPs) as well as pentraxin-3 (PTX-3) were then quantified using a multiplex immunoassay. Our results revealed a striking functional dichotomy. Under normoxic conditions, CAPE suppressed the secretion of key pro-inflammatory mediators. Conversely, under hypoxic conditions, CAPE significantly amplified the release of pro-tumorigenic factors, including the mediator facilitating tumor cell migration, invasion, and angiogenesis such as IL-8 and the invasion-associated metalloproteinase MMP-2. These findings suggesting that hypoxia may fundamentally reprograms the immunomodulatory potential of CAPE. However, due to limitations of study requires further validation in a broader panel of glioblastoma models.

## 1. Introduction

**Figure 1:**
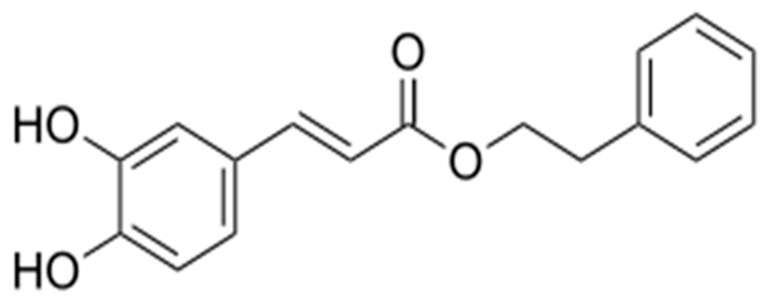
Structure of CAPE.

To address the pivotal role of inflammation within the glioblastoma multiforme (GBM) microenvironment, agents with potent immunomodulatory activity have attracted increasing scientific attention. One such compound is caffeic acid phenethyl ester (CAPE), a bioactive component of propolis recognized for its pleiotropic anti-inflammatory and anti-neoplastic properties. CAPE was first synthesized in 1988 at Columbia University. The chemical structure of CAPE is shown in Figure 1. The hydroxyl groups within the catechol moiety of CAPE confer its multifunctional biological activities, including antioxidant, antiviral, antibacterial, anti-inflammatory, anti-cancer, and immunomodulatory effects [1,2,3,4,5].

CAPE modulates inflammatory pathways primarily through inhibition of the nuclear factor kappa B (NF-κB) signaling cascade. NF-κB plays a central role in immune regulation, cell proliferation, and inflammation. Pro-inflammatory cytokines such as IL-1, IL-6, TNF-α, and adhesion molecules activate NF-κB. CAPE suppresses NF-κB activation by preventing its DNA-binding capacity [6,7]. Furthermore, CAPE exhibits pro-apoptotic properties by enhancing TRAIL- and FAS-mediated apoptosis in cancer cells [8].

CAPE also interferes with angiogenesis by downregulating vascular endothelial growth factor (VEGF) signaling [9,10,11].

Despite its established therapeutic potential, the bioactivity of CAPE within the complex metabolic landscape of the GBM core is poorly understood. While some studies suggest CAPE can counteract hypoxia-driven processes such as angiogenesis by suppressing VEGF, these observations were predominantly made in non-glioma models. The severely hypoxic and inflammatory milieu of GBM imposes unique selective pressures that can fundamentally alter cellular responses and drug efficacy. Therefore, a critical and unanswered question remains: does CAPE retain its anti-inflammatory functions within the GBM microenvironment, or does hypoxia trigger a functional conversion, potentially transforming a therapeutic agent into a modulator of a pro-tumorigenic type? Hypoxia occurs particularly in solid malignancies. Under low-oxygen conditions, cancer cells undergo metabolic reprogramming that stimulates angiogenesis and erythropoiesis. To adapt, they upregulate glucose transport and glycolytic flux and engage in de novo fatty-acid synthesis [12].

Moreover, CAPE impacts on tumor metastasis inhibiting expression of metalloproteinases (MMPs). Therefore, CAPE, by its effects on angiogenesis, metastasis regulates the tumor microenvironment.

Additionally, CAPE inhibits cyclooxygenase-2 (COX-2) activity and exerts the impact on metastasis and proliferation. Further, chronic inflammation can promote the development of cancer by damaging cell DNA, RNA stimulating cell division, inhibiting apoptosis and promoting angiogenesis. CAPE exhibits anti-tumor activity through inhibition of DNA synthesis, disruption of growth signaling, induction of apoptosis, and anti-angiogenic properties [13,14,15]. Chang et al. demonstrated that CAPE at concentration of 25 µg/mL inhibited proliferation and migration in MDA-MB-231 breast cancer followed by LPS stimulation [16]. Due to strong biological activities, CAPE seems to be valuable adjuvant in therapeutic applications especially for anti-cancer therapy and chemoprevention. CAPE treatment enhances the anticancer therapy of doxorubicin, vinblastine, paclitaxel, estramustine, docetaxel, 5-fluorouracil, tamoxifen [3,17]. Conducted studies confirm that CAPE may be considered as adjuvant in therapy of: prostate, gastric, lung, head and neck as well as breast cancer [9,18,19]. Given its ability to cross the blood–brain barrier, CAPE represents a viable clinical candidate. Accordingly, the primary objective of this study was to elucidate the impact of hypoxia on the immunomodulatory activity of CAPE in a human astrocytoma cell model. We specifically aimed to profile the secretion of key pro- and anti-inflammatory cytokines and matrix metalloproteinases under normoxic versus hypoxic conditions to determine whether oxygen concentration acts as a functional switch for CAPE’s bioactivity in the context of glioblastoma. Pilocytic astrocytomas (Grade I) according to the World Health Organization (WHO) classification are usually benign tumors that occur mainly in children and are generally associated with favorable clinical outcomes. In contrast, diffuse gliomas—including astrocytomas, oligodendrogliomas, and oligoastrocytomas—are graded as either low-grade (Grade II) or high-grade (Grades III and IV), reflecting their biological behavior and clinical prognosis. Importantly, high-grade gliomas demonstrate a marked tendency toward progression and malignant transformation, frequently culminating in the development of glioblastoma (GB), which is classified as a Grade IV astrocytoma. GBM, according to a WHO Grade IV astrocytoma, stands as the most lethal primary malignancy of the central nervous system, notorious for its therapeutic recalcitrance and dismal prognosis. The pathobiology of GBM is intimately linked to its unique tumor microenvironment (TME), a complex ecosystem governed by two interdependent pillars: chronic inflammation and severe hypoxia. This hypoxic-inflammatory axis is not merely a feature of GBM but a principal driver of tumor progression, invasion, and resistance to standard radio- and chemotherapy, thus representing a critical target for novel therapeutic interventions [19]. Due to the ability of CAPE contained in propolis to cross the blood–brain barrier (BBB) and the immunomodulatory effect of propolis on highly malignant adult diffuse glioma cells, which we have previously investigated, CAPE shows potential as an anti-glioma agent. The aim of the present study was to evaluate the impact of CAPE on concentration of selected cytokines such as interleukin (IL) such as IL-8, IL-26, IL-10, metalloproteinases (MMP-1, -2 -3), and pentraxin-3 (PTX-3) which are produced by astrocytes of the CCF-STTG1 cell line followed by stimulation of LPS and/or interferon-α. According to the best of our knowledge, this study provides the first comprehensive evaluation of CAPE-mediated modulation of cytokines (IL-8, IL-26, IL-10), PTX3, and metalloproteinases (MMP-1/-2/-3) in LPS- and/or IFN-α–stimulated CCF-STTG1 astrocytes, establishing an anti-glioma immunomodulatory potential of CAPE.

## 2. Results

The conducted study was designed to determine the impact of CAPE on the selected cytokines and metalloproteinases as well as PTX-3, which are connected with tumor microenvironment and metastasis.

### 2.1. Effect of CAPE on the Viability of CCF-STTG1 Human Astrocytoma Cells Followed by Stimulation of LPS and/or IFN-α in Normoxic and Hypoxic Conditions

**Figure 2:**
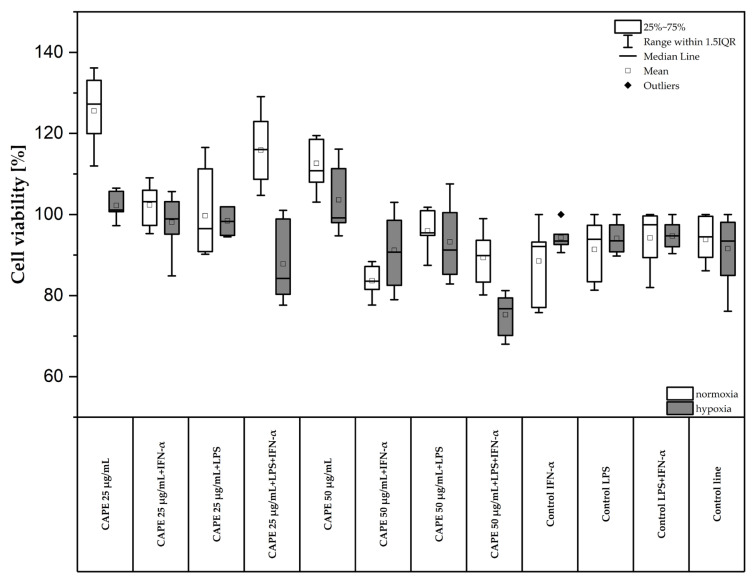
Viability of CCF-STTG1 cells treated with CAPE, LPS, and IFN-α under normoxia (white boxes) and hypoxia (gray boxes) evaluated by the MTT assay (*n* = 3). Results are expressed as percentage of control viability (mean ± SD). The box plots show the median (horizontal line), mean (□ symbol), and interquartile range (IQR). Whiskers represent the range of values excluding outliers.

The viability of CCF-STTG1 cell line after the application of CAPE was determined using the MTT assay (3-[4,5-dimethylthiazol-2-yl]-2,5-diphenyltetrazolium bromide). CAPE was tested at two concentrations: 25 µg/mL (87.93 µM) and 50 µg/mL (175.86 µM). The results are presented in Figure 2. Under normoxic conditions, the greatest reduction in cell viability was observed for CAPE at concentration of 50 µg/mL (175.86 µM) following combined LPS + IFN-α stimulation. Under normoxia, CAPE at concentration of 50 µg/mL (175.86 µM) decreased cell viability from 94.80 ± 3.05% to 75.61 ± 4.51% (Figure 2). Under hypoxic conditions, the most pronounced decrease in viability was recorded for CAPE treatment combined with IFN-α stimulation. Cell viability decreased from 88.60 ± 8.25% to 83.16 ± 3.63% (Figure 2). Similarly, followed by LPS + IFN-α stimulation under hypoxia, CAPE (50 µg/mL) decreased cell viability from 91.02 ± 8.89% to 89.73 ± 5.97%.

According to ISO 10993-5:2009, a reduction in cell viability below 70% relative to the untreated control is considered indicative of cytotoxicity in the MTT assay [20]. In the present study, CAPE at 25 and 50 µg/mL did not reduce CCF-STTG1 viability below 70%, and therefore was classified as non-cytotoxic according to ISO criteria. Consequently, these concentrations were selected for subsequent experiments as the highest non-cytotoxic doses, aimed at maximizing the sensitivity of cytokine detection in this short-term in vitro model.

### 2.2. Effect of CAPE on the Secretion of Selected Cytokines by the CCF-STTG1 Human Astrocytoma Cell Line Following LPS and/or IFN-α Stimulation in Normoxic and Hypoxic Conditions

**Figure 3:**
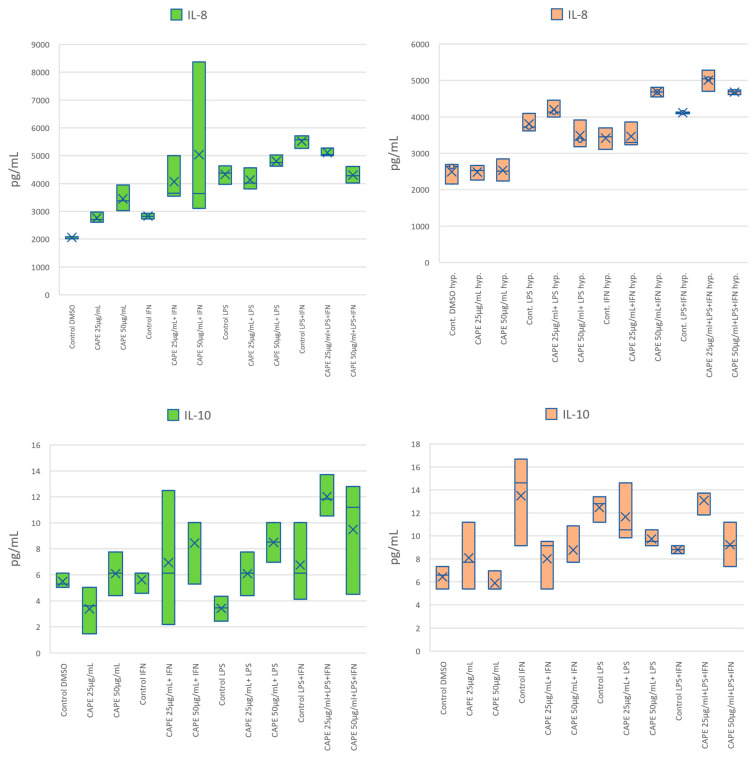

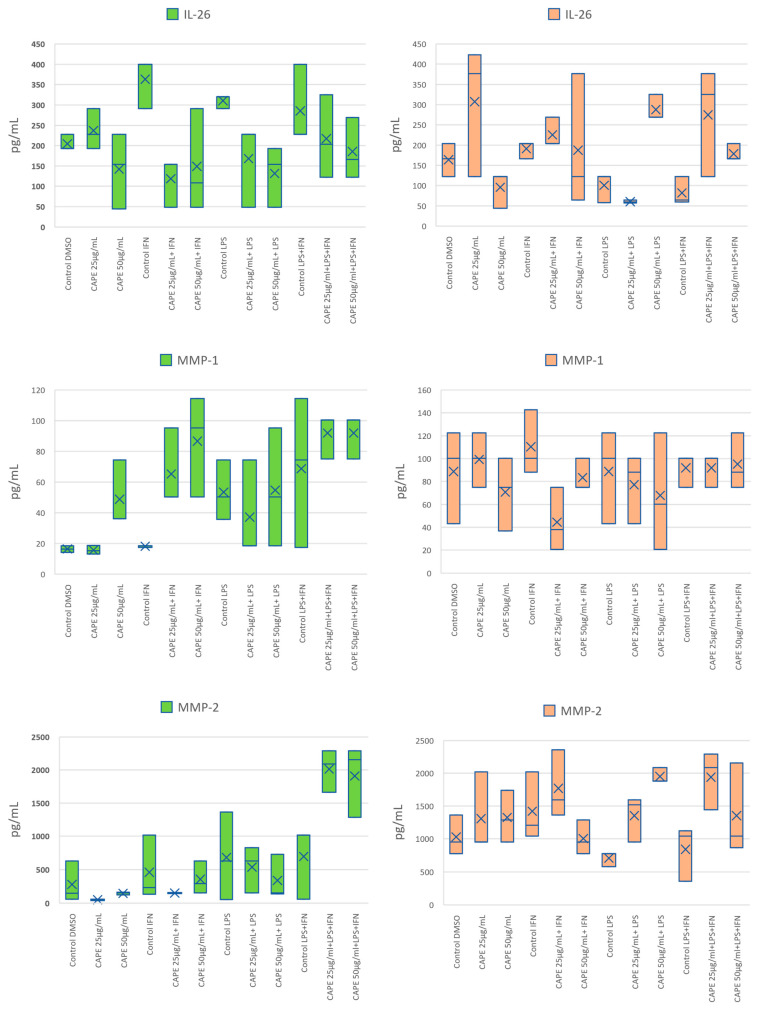

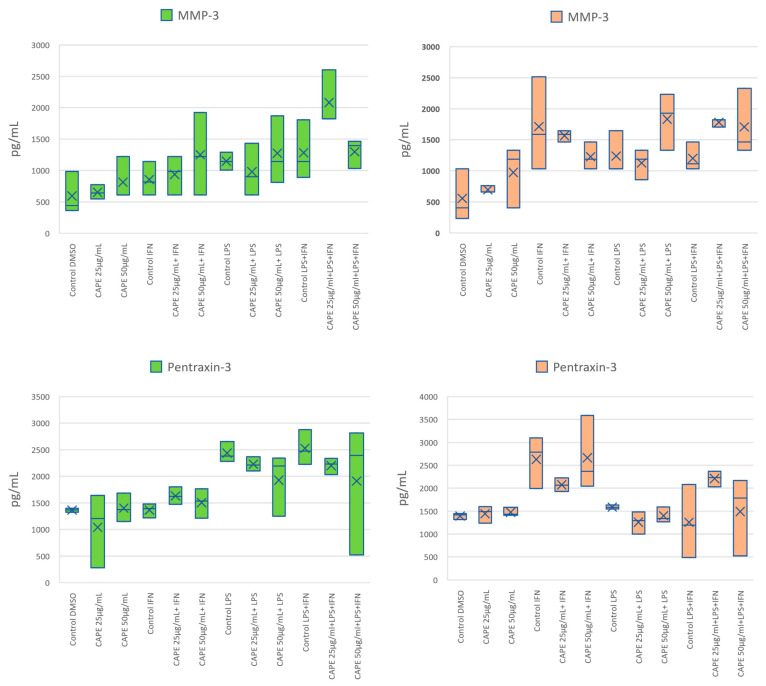
The effect of CAPE on selected cytokines: IL-8, IL-10, IL-26, metalloproteinases MMP-1, -2, -3 and PTX3 by native and stimulated astrocytes cell line CCF-STTG1 by LPS or/and IFN-α in normoxic (left figures) and hypoxic (right figures) conditions. Data are expressed as mean ± SD (pg/mL) derived from three independent biological replicates (n = 3). Boxes represent the interquartile range (IQR), with the horizontal line indicating the median and the ‘x’ marking the mean. Whiskers show the minimum and maximum values (excluding outliers). Green and orange colors denote data from normoxic and hypoxic conditions, respectively.

The influence of CAPE on the secretion of cytokines (IL-8, IL-10, IL-26), metalloproteinases (MMP-1, -2, -3), and pentraxin-3 (PTX3) was assessed in CCF-STTG1 astrocytoma cells stimulated with LPS and/or IFN-α under normoxic and hypoxic conditions. The results are presented in Figure 3 and in Table 1. LPS and IFN-α stimulation significantly increased cytokine secretion in control CCF-STTG1 cells.

Under normoxia, CAPE at concentration of 50 µg/mL (175.86 µM) significantly increased IL-8 secretion in IFN-α-stimulated cells compared with the respective control (*p* = 0.006). In other stimulatory models, CAPE at concentrations of 25 or 50 µg/mL (87.93 µM or 175.86 µM) did not produce statistically significant changes in IL-8 levels. Under hypoxia, CAPE did not significantly alter IL-8 secretion in any of the stimulation settings tested.

Regarding IL-10, under normoxia CAPE at 50 µg/mL (175.86 µM) significantly in-creased IL-10 secretion following LPS stimulation compared with the matched control (*p* = 0.0228). Following LPS + IFN-α co-stimulation, CAPE at 25 µg/mL (87.93 µM) also increased IL-10 levels (*p* = 0.0189). Under hypoxia, CAPE decreased IL-10 in LPS-stimulated cells at both 25 and 50 µg/mL compared with the matched LPS control (*p* = 0.0026 and *p* = 0.0078, respectively). Conversely, under hypoxia with LPS + IFN-α co-stimulation, CAPE at 25 µg/mL increased IL-10 relative to the matched control (*p* = 0.0145), whereas 50 µg/mL showed no significant effect. Following LPS + IFN-α stimulation, CAPE at 25 µg/mL significantly increased IL-10 levels (*p* = 0.014536), whereas no significant effect was observed at 50 µg/mL.

CAPE treatment at both concentrations reduced IL-26 secretion following either IFN-α or LPS stimulation under normoxia. At concentrations of 25 and 50 µg/mL CAPE significantly suppressed IL-26 levels in IFN-α– and LPS-stimulated cells (*p* = 0.0010–0.0119). Both CAPE at concentration of 25 µg/mL (87.93 µM) and CAPE at concentration of 50 µg/mL (175.86 µM) followed by LPS stimulation, significantly decreased in IL-26 levels compared to the control group stimulated with LPS (*p* = 0.0404 and *p* = 0.0119), respectively.

Under hypoxia, CAPE alone or combined with LPS had no significant effect on IL-26. However, IL-26 secretion was significantly increased by CAPE at concentration of 50 µg/mL (175.86 µM) followed by IFN-α stimulation, *p* = 0.0105) and CAPE at concentration of 25 µg/mL (87.93 µM) followed by LPS + IFN-α stimulation, *p* = 0.0087), indicating a concentration- and stimulus-dependent response (Figure 3). Collectively, these results indicate an oxygen-dependent shift in CAPE’s cytokine modulation: under normoxia, CAPE increased IL-10 and decreased IL-26, whereas under hypoxia CAPE increased IL-26 in selected stimulation settings, while IL-8 was not significantly modified.

Under normoxic conditions, CAPE significantly modulated MMP-1 expression. Following IFN-α stimulation, CAPE at both concentrations of 25 µg/mL (87.93 µM; *p* = 0.0347) and50 µg/mL (175.86 µM; *p* = 0.0033) significantly increased MMP-1 levels compared with the respective control. This effect was not reproduced under hypoxia. In contrast, a significant decrease in MMP-1 concentration was observed under hypoxic conditions in cells treated with CAPE at concentration of 25 µg/mL (87.93 µM) combined with LPS stimulation (*p* = 0.0148) relative to the corresponding control group.

For MMP-2, CAPE produced no statistically significant changes under normoxia when applied alone or in combination with single LPS or IFN-α stimulation. However, following combined LPS + IFN-α stimulation, CAPE further increased MMP-2 secretion at both concentrations of 25 µg/mL (87.93 µM; *p* = 0.0003) and 50 µg/mL (175.86 µM; *p* = 0.0007). Under hypoxic conditions, CAPE alone or in the presence of LPS exerted no significant effect on MMP-2 levels. Notably, the stimulatory effect of CAPE on MMP-2 production was observed only in IFN-α-treated cells and depended both on concentration and co-stimulation pattern: in the presence of IFN-α alone, only at concentration of 50 µg/mL (175.86 µM) CAPE increased MMP-2 secretion (*p* = 0.0018), while under LPS + IFN-α co-stimulation, a significant effect was detected exclusively for the lower concentration (25 µg/mL equal to 87.93 µM; *p* = 0.0048).

Regarding MMP-3, a statistically significant decrease (*p* = 0.0191) was observed under normoxia in cells treated with CAPE at concentration of 25 µg/mL (87.93 µM) following LPS + IFN-α stimulation compared with the respective control. No significant changes in MMP-3 levels were detected at higher CAPE concentrations or under other stimulation models. Similarly, under hypoxia, CAPE did not significantly alter MMP-3 secretion under any tested condition.

CAPE treatment did not significantly affect PTX3 levels under most experimental conditions. All *p*-values obtained for comparisons between treated and control groups exceeded the α = 0.05 threshold, indicating no statistically supported modulation of PTX3 by CAPE. The only exception was a significant increase (*p* = 0.0181) in PTX3 concentration under hypoxia in cells treated with CAPE at concentration of 25 µg/mL (87.93 µM) following LPS + IFN-α stimulation.

Collectively, these findings indicate that CAPE regulates MMP expression in a context- and oxygen-dependent manner. Under normoxia, CAPE enhances MMP-1 and MMP-2 secretion predominantly in IFN-α–activated cells, while under hypoxia, its effect shifts toward suppression of MMP-1 and selective activation of PTX3. Such dual modulation suggests a potential reprogramming of extracellular matrix remodeling by CAPE within the hypoxic glioblastoma microenvironment.

### 2.3. Statistical Analysis of CAPE’s Effects on Cytokine, Metalloproteinase, and Pentraxin-3 Secretion by CCF-STTG1 Astrocytoma Cells Under Normoxic and Hypoxic Conditions

Based on the results of the conducted analyses, CAPE did not exhibit any statistically significant effects at either tested concentration on the basal secretion of interleukins, extracellular matrix metalloproteinases, or PTX-3.

Under normoxic conditions, IL-8 levels in cells treated with CAPE at concentration of 25 µg/mL (mean = 2761.03 ± 191.21) and at concentration of 50 µg/mL (mean = 3451.08 ± 470.63) did not differ significantly from the DMSO control group (mean = 2062.81 ± 38.18; *p* = 0.353 and *p* = 0.072, respectively). Similarly, no significant differences were observed for IL-10 (*p* = 0.323 for 25 µg/mL; *p* = 0.171 for 50 µg/mL) or IL-26 *(p* = 0.622 for 25 µg/mL; *p* = 0.351 for 50 µg/mL) compared with the control.

CAPE treatment did not affect basal metalloproteinase secretion. Levels of MMP-1, MMP-2, and MMP-3 measured in CAPE-treated cells (25 and 50 µg/mL) did not significantly differ from those in the DMSO control (all *p* > 0.05). Similarly, CAPE at both concentrations did not modulate basal PTX3 secretion (*p* = 0.415 and *p* = 0.922, respectively).

These results clearly demonstrate that under basal conditions—without pro-inflammatory stimulation—CAPE does not alter the secretion of IL-8, IL-10, IL-26, MMP-1, MMP-2, MMP-3, or PTX3. This indicates that, in the examined astrocytoma model, CAPE does not act per se but functions as a modulator of cell responses to inflammatory stimuli, as further analyzed in subsequent experiments. Prior to evaluating CAPE, we analyzed the basal effect of oxygen deprivation. Comparisons between vehicle controls (Control DMSO) revealed that hypoxia alone significantly upregulated the basal secretion of matrix metalloproteinases compared to normoxia. Specifically, MMP-2 levels rose from 278.2 ± 307.6 pg/mL (normoxia) to 1029.8 ± 303.1 pg/mL (hypoxia), and MMP-1 levels increased from 16.3 ± 2.1 pg/mL to 88.7 ± 41.0 pg/mL (Table 1). This confirms that the experimental conditions successfully induced a hypoxia-responsive phenotype in CCF-STTG1 cells, consistent with HIF-1α-mediated extracellular matrix remodeling. Under hypoxic conditions, a pronounced shift in CAPE’s activity profile was observed, with the compound predominantly enhancing the pro-inflammatory response. The experimental conditions exerted a highly significant overall effect on the biomarker panel (Wilks’ λ test, F(77, 115.3) = 3.518, *p* < 0.0001).

CAPE alone did not affect basal IL-8 secretion; however, in the presence of inflammatory stimuli, it acted as an enhancer of IL-8 production. A statistically significant increase in IL-8 was observed for CAPE at concentration of 25 µg/mL with IFN-α (mean = 4196.43 ± 238.10 vs. control 3418.28 ± 295.71; *p* = 0.001131), for CAPE at concentration of 50 µg/mL with LPS stimulation (mean = 4681.25 ± 134.01 vs. control 3812.95 ± 249.14; *p* = 0.000385), and for both concentrations following LPS + IFN-α co-stimulation (*p* = 0.0003 for 25 µg/mL and *p* = 0.0124 for 50 µg/mL).

The effect of CAPE on IL-10 secretion was context-dependent. Following LPS stimulation, both CAPE at concentrations of 25 and 50 µg/mL significantly reduced IL-10 levels (means = 8.01 ± 2.30 and 8.77 ± 1.81, respectively, vs. control 13.48 ± 3.88; *p* = 0.002560 and *p* = 0.007808). In contrast, after LPS + IFN-α co-stimulation, CAPE at 25 µg/mL significantly increased IL-10 secretion (mean = 13.08 ± 1.09 vs. control 8.80 ± 0.36; *p* = 0.0145).

CAPE exerted a stimulatory effect on IL-26 secretion. A significant increase was observed for CAPE at concentration of 50 µg/mL following IFN-α stimulation (mean = 287.66 ± 32.63 vs. control 100.48 ± 37.18; *p* = 0.0105) and for CAPE at concentration of 25 µg/mL after LPS + IFN-α stimulation (mean = 274.54 ± 134.59 vs. control 81.92 ± 34.75; *p* = 0.0087).

Regarding matrix metalloproteinases, a single suppressive effect was identified: CAPE at concentration of 25 µg/mL following LPS stimulation significantly decreased MMP-1 levels (mean = 44.50 ± 27.73 vs. control 110.42 ± 28.66; *p* = 0.0148). No other significant effects were detected.

In contrast, CAPE enhanced MMP-2 production under specific stimulatory conditions. A significant increase was noted for CAPE at concentration of 50 µg/mL following IFN-α stimulation (mean = 1950.72 ± 120.08 vs. control 707.67 ± 112.49; *p* = 0.001792) and for CAPE 25 µg/mL after LPS + IFN-α stimulation (mean = 1940.57 ± 444.03 vs. control 840.25 ± 419.05; *p* = 0.004795). For MMP-3, no statistically significant changes were observed under any condition.

With respect to PTX3, a single significant increase was detected for CAPE at concentration of 25 µg/mL following LPS + IFN-α stimulation (mean = 2210.82 ± 167.09 vs. control 1256.76 ± 798.91; *p* = 0.0181)

Taken together, these results demonstrate that under hypoxic conditions, the data suggest an oxygen-dependent shift in CAPE-associated response patterns acting primarily as an enhancer of pro-inflammatory mediators while only occasionally exerting suppressive effects. In light of these findings, more advanced statistical methods, including principal component analysis (PCA) and hierarchical clustering analysis (HCA), were applied to further elucidate the interrelationships among the measured variables.

**Figure 4:**
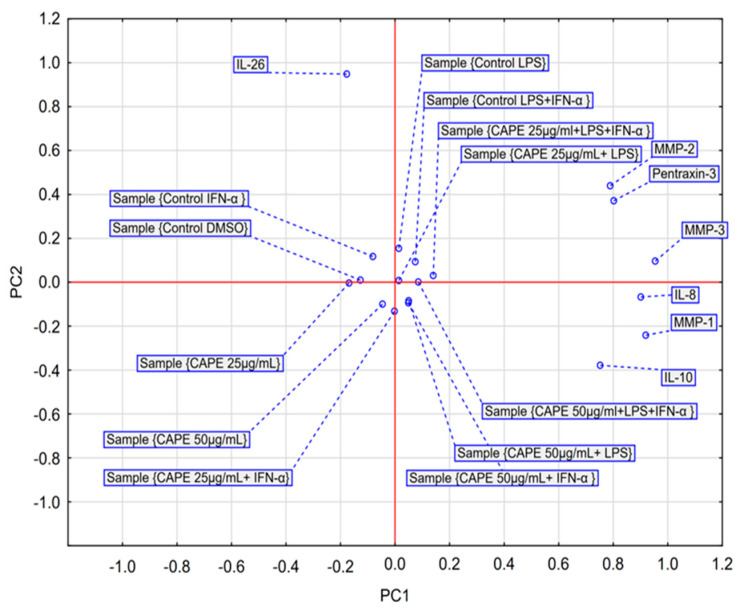
PCA score plot of all obtained data based on the average content of the effect of CAPE at both concentrations (25 and 50 μg/mL) in normoxic conditions on selected cytokines, metalloproteinases and PTX-3. PC—principal component.

The PCA score plot was generated to visualize the average effects of two CAPE concentrations on the secretion of selected markers under normoxic conditions (Figure 4). The first two principal components (PC1 and PC2) together explained 75.73% of the total variance in the dataset (R^2^X = 0.757), while maintaining adequate predictive power, as indicated by the cross-validation parameter (Q^2^ = 0.438). PC1, which accounted for 56.54% of the variance, clearly separated the samples according to LPS stimulation status. This component was strongly and positively correlated with MMP-1, MMP-2, MMP-3, IL-8, IL-10, and PTX3 levels. LPS-stimulated groups were positioned on the positive side of the PC1 axis, whereas non-LPS-stimulated groups clustered on the negative side. Accordingly, PC1 represents the principal dimension of the pro-inflammatory response driven by LPS. PC2 explained 19.20% of the total variance and was predominantly influenced by interleukin-26 (IL-26) loadings. This component effectively discriminated the control groups (Control DMSO and Control IFN-α), characterized by high IL-26 levels (positive values), from the CAPE-treated groups, which exhibited markedly lower IL-26 secretion (negative values). Thus, PC2 reflects the axis of variability associated with the suppressive effect of CAPE on IL-26 production.

The PCA scatter plot revealed three well-defined clusters corresponding to distinct biochemical phenotypes:

Resting/IFN-α-dependent profile—including Control DMSO and Control IFN-α groups, located in the upper left quadrant, characterized by elevated IL-26 and the absence of LPS-driven responses.

CAPE-suppressed profile—encompassing CAPE 25 µg/mL, CAPE 50 µg/mL, and CAPE 25 µg/mL combined with IFN-α stimulation, forming a compact cluster in the lower left quadrant that represents the anti-inflammatory activity of CAPE, primarily mediated through IL-26 suppression.

LPS-response profile—comprising all LPS-stimulated groups, irrespective of CAPE treatment, grouped on the right side of the plot. This cluster illustrates that the pro-inflammatory signature induced by LPS dominates the overall biochemical variance, rendering CAPE’s modulatory impact secondary in defining this phenotype.

In summary, PCA demonstrated that under normoxic conditions, CAPE was associated with a shift in the global biochemical profile of glioblastoma cells in the absence of LPS stimulation, primarily through suppression of IL-26. Conversely, under LPS exposure, the cellular response remains largely coordinated and LPS-dominated, diminishing the relative contribution of CAPE’s modulatory effects.

**Figure 5:**
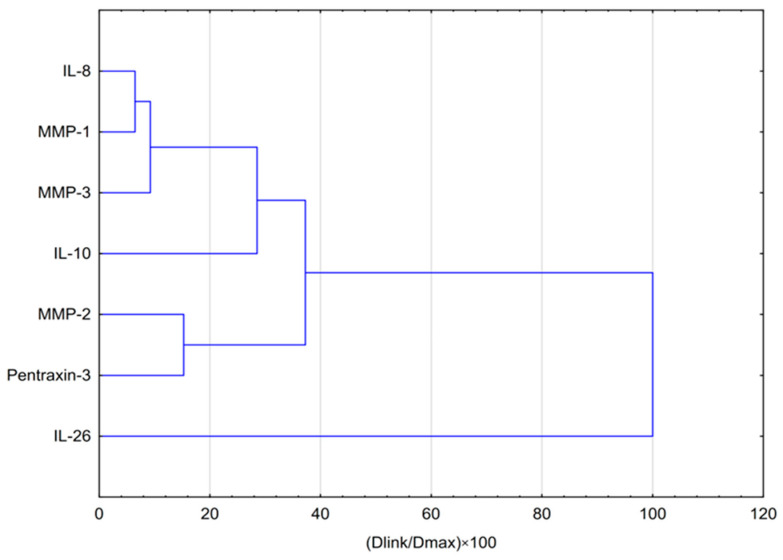
Dendrogram obtained via HCA of data regarding the behavior of the effect of CAPE at both concentrations (25 and 50 μg/mL) in normoxic conditions on selected cytokines, metalloproteinases and PTX-3. Dlink denotes the linkage distance, defined as the quantitative measure of separation between two clusters, whereas Dmax represents the maximum attainable distance between any pair of clusters.

The dendrogram obtained from the hierarchical cluster analysis (HCA) (Figure 5) corroborates and extends the conclusions drawn from the PCA, visualizing the functional interrelationships among the cytokines and matrix-associated proteins evaluated under normoxic conditions. The analysis revealed several distinct and biologically coherent clusters.

At the highest level of agglomeration, the data structure exhibited a fundamental division that separated interleukin-26 (IL-26) from all other mediators. This finding confirms the unique regulatory profile of IL-26, previously identified as the primary contributor to the second principal component (PC2). The remaining six biomarkers—IL-8, IL-10, MMP-1, MMP-2, MMP-3, and PTX3—formed a second major cluster representing a coordinated pro-inflammatory network corresponding to the first principal component (PC1). Further examination of this cluster’s internal organization revealed two well-defined subgroups: one comprising MMP-2 and PTX3, and the other consisting of IL-8, MMP-1, MMP-3, and IL-10. This subdivision suggests the existence of at least two tightly interconnected axes of biochemical response within the stimulation-driven pro-inflammatory reaction—one reflecting extracellular matrix remodeling (MMP-2/PTX3 axis) and the other associated with cytokine-mediated signaling (IL-8/IL-10/MMP-1/MMP-3 axis).

**Figure 6:**
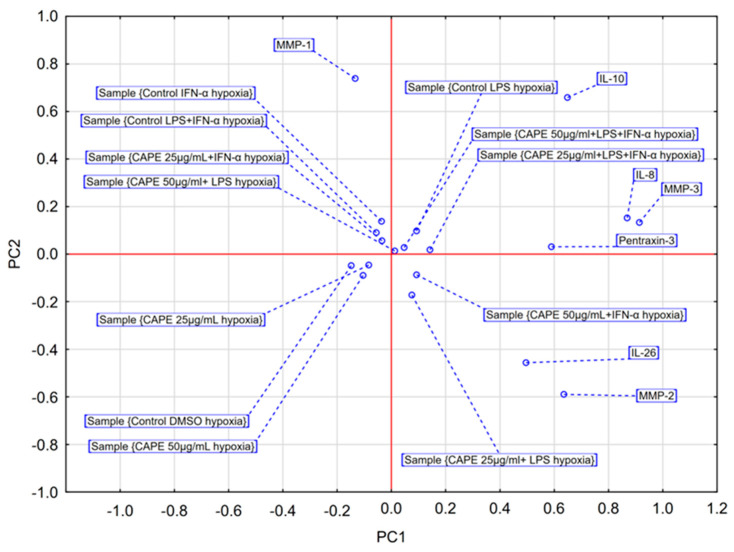
PCA score plot of all obtained data based on the average content of the effect of CAPE at both concentrations (25 and 50 μg/mL) in hypoxic conditions on selected cytokines, metalloproteinases and PTX-3. PC—principal component.

The PCA score plot was generated to assess the average effects of two CAPE concentrations on the secretion of selected markers under hypoxic conditions (Figure 6). The PCA model explained 59.83% of the total variance across the first two components (PC1 = 38.97%, PC2 = 20.86%). However, the model did not meet strict cross-validation criteria (Q^2^ for PC1 = 0.100; Q^2^ for PC2 = −0.100), and only PC1 reached statistical significance. Therefore, interpretation primarily focused on stable variance patterns along PC1, whereas variation along PC2 was treated with caution as potentially unstable.

The analysis demonstrated that hypoxia profoundly reconfigures the network of cellular responses. In contrast to the normoxic model, which was dominated by a single LPS-driven response axis, PC1 under hypoxia delineated two divergent inflammatory profiles (Figure 6). PC1 exhibited strong positive correlations with IL-8, MMP-3, IL-10, and PTX3, clustering the LPS-stimulated control group and related samples on the positive (right) side of the plot. Conversely, PC1 showed negative correlations with MMP-1, forming a distinct cluster on the negative (left) side of the axis that encompassed all IFN-α–stimulated groups (alone or in co-stimulation).

The scatter plot confirmed this dichotomy, revealing a clear spatial separation between two dominant response clusters: an “IFN-α–dominated profile” on the left and a broader “LPS-associated pro-inflammatory profile” on the right. Notably, the unstimulated samples (Control DMSO, CAPE 25 µg/mL, and CAPE 50 µg/mL) formed a compact, discrete cluster in the center of the plot, indicating that CAPE alone does not substantially alter the global biochemical profile of astrocytoma cells under hypoxia.

In summary, despite limitations in model validation, PCA under hypoxic conditions provides an integrated multivariate view of the measured readout. Hypoxia polarizes the inflammatory response into at least two distinct biochemical phenotypes: one IFN-α–driven, characterized by robust MMP-1 induction, and the other LPS-associated, encompassing a broader set of pro-inflammatory mediators including IL-8, MMP-3, and PTX3. The role of CAPE under hypoxia appears confined to modulation within these pre-existing phenotypes rather than the induction of a novel regulatory pattern.

**Figure 7:**
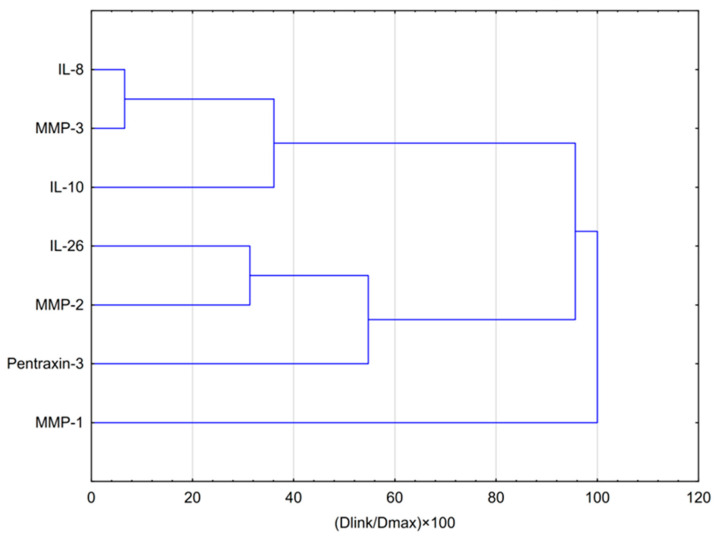
Dendrogram obtained via HCA of data regarding the behavior of the effect of CAPE at both concentrations (25 and 50 μg/mL) in hypoxic conditions on selected cytokines, metalloproteinases and PTX-3. Dlink denotes the linkage distance, defined as the quantitative measure of separation between two clusters, whereas Dmax represents the maximum attainable distance between any pair of clusters.

The results of the hierarchical cluster analysis (HCA) (Figure 7) fully corroborated and extended the PCA findings, confirming a profound reorganization of cellular response patterns under hypoxia. The dendrogram revealed that MMP-1 exhibited the most distinct variability profile, consistent with its role as a defining marker of the separate response axis identified along the negative values of PC1. The remaining six biomarkers formed a second major cluster characterized by additional internal structure. Within this cluster, two subgroups were clearly distinguishable: the first included IL-8 and MMP-3, which were closely associated with IL-10, while the second comprised MMP-2 and PTX3, strongly linked with IL-26.

These findings quantitatively confirm that hypoxia not only reshapes the overall architecture of cellular responses but also polarizes it into at least three distinct, hierarchically organized groups of co-regulated mediators, reflecting divergent functional programs within the hypoxic glioblastoma microenvironment.

## 3. Discussion

In this study, we demonstrated the effects of caffeic acid phenethyl ester (CAPE) on the release of selected cytokines, matrix metalloproteinases, and adhesion molecules by the human astrocytoma cell line CCF-STTG1 following LPS and/or IFN-α stimulation under both normoxic and hypoxic conditions. In addition, we evaluated the cytotoxicity of CAPE in the same experimental settings.

Our findings revealed that CAPE decreased the viability of CCF-STTG1 cells only at the highest tested concentration, both under normoxia and hypoxia. Under normoxic conditions, cytotoxicity was most pronounced following LPS + IFN-α co-stimulation. Under hypoxia, the strongest reduction in viability was observed after CAPE treatment combined with IFN-α or LPS + IFN-α stimulation. These results are consistent with the study by Morin et al. [21], who reported significant cytotoxicity of CAPE against glioma cell lines Hs683 and LN319. Similarly, Kabała-Dzik et al. demonstrated that CAPE exhibits cytotoxic activity against the triple-negative breast adenocarcinoma cell line MDA-MB-231 at concentrations of 50 µM and 100 µM [22]. Furthermore, Borawska et al. reported that the combination of *Hypericum perforatum* L. and ethanolic extract of propolis produced a time- and dose-dependent reduction in the viability of glioblastoma multiforme U87MG cells [23].

Glioblastoma remains one of the most aggressive and therapeutically challenging brain tumors, characterized by extensive infiltration, rapid proliferation, and poor prognosis. Persistent inflammation represents a defining feature of its pathophysiology, shaping the tumor microenvironment to support proliferation, invasion, angiogenesis, and immune evasion [24]. Glioblastoma is a highly malignant primary brain tumor characterized by markedly heterogeneous intratumoral oxygenation. Physiologically, oxygen tension within glioblastoma (GBM) tissue is markedly lower than atmospheric levels. Several studies have reported that GBM exhibits intra-tumoral oxygen tensions ranging between 0.5% and 5% oxygen, with the region deep inside a solid tumor often falling below 1% oxygen. Thus, our chosen oxygen concentration of 1% falls within the physiologically relevant hypoxic range observed in GBM tumors. We selected this condition based on the following considerations. Our chosen hypoxic level reflects oxygen tensions measured in the hypoxic regions of GBM, thereby providing a realistic in vitro approximation of the tumor microenvironment. The majority of GBM hypoxia studies employ oxygen concentrations between 0.5% and 2% oxygen, enabling us to compare our findings with previously published work and ensure methodological consistency. Moreover, at this oxygen level, GBM cells reliably activate hypoxia-responsive pathways (e.g., HIF-1α stabilization), supporting its suitability for studying molecular mechanisms induced by hypoxia [25,26,27].

In recent years, the complexity and heterogeneity of the tumor microenvironment has become increasingly apparent, highlighting its key role in shaping tumor behavior and response to treatment. Various components of the TME—including immune infiltration, stroma cells and extracellular matrix dynamics—collectively influence tumor progression and treatment resistance. Comprehensive TME profiling has emerged as a strategy to understand tumor–immune interactions and identify therapeutic vulnerabilities [28,29]. Recent advances in the systematic study of TME and nanomaterial-based immunomodulatory approaches further reinforce this direction, offering promising tools to enhance anti-tumor immunity in the heterogeneous tumor microenvironment ([30]). Consideration of these insights places our results within a broader framework of TME-oriented therapeutic innovation. Metabolic reprogramming is another critical feature closely linked to TME remodeling and malignant progression. The observations of our study are consistent with reports that metabolic plasticity enables cancer cells to adapt to nutrient fluctuations, evade immune surveillance and develop drug resistance. State-of-the-art metabolic analysis technologies have improved our understanding of the complex metabolic pathways that regulate the adaptation of tumor cells to the tumor microenvironment and the mechanisms that promote progression and resistance to treatment. In particular, recent studies on metabolic reprogramming associated with the mevalonate pathway highlight its integral role in supporting tumor proliferation and survival [31]. These findings reinforce the rationale for targeting metabolic vulnerabilities as a complementary strategy to conventional therapies. Given the increasing emphasis on precision therapies, the implications of our findings extend to the discovery of adjuvants that act in the tumor microenvironment. Glioma—one of the most metabolically aggressive and treatment-resistant malignancies—has been the subject of numerous drug design studies aimed at disrupting oncogenic signaling and metabolic dependencies. In the present work, we focused on the influence of CAPE on both pro- and anti-inflammatory cytokines in astrocytoma cells. Our results indicate that CAPE’s impact on inflammatory and tissue-remodeling mediators differs fundamentally between normoxic and hypoxic conditions. Under normoxia, CAPE functioned as a context-dependent anti-inflammatory modulator. In physiological oxygen conditions, its behavior was largely consistent with its well-established anti-inflammatory profile, although its activity was strictly dependent on the nature of the applied stimulation. Taken together, our data suggest that while CAPE exhibits modulatory activity within the glioma microenvironment, it may also enhance certain pro-inflammatory cytokines and metalloproteinases under specific conditions. These observations, although requiring confirmation in other glioma models, imply that CAPE does not uniformly exert a beneficial influence on the tumor microenvironment and may, in hypoxic contexts, promote a shift toward a pro-tumorigenic phenotype.

Interleukin-8 (IL-8), also known as cysteine-X-cysteine (CXC) motif chemokine ligand 8 (CXCL8), is a pro-inflammatory cytokine belonging to the CXC chemokine family. It is released by endothelial and epithelial cells, immune cells, and various cancer cell types [27]. According to Meier and Brieger [32], IL-8 supports tumor cell proliferation through both autocrine and paracrine mechanisms. Furthermore, IL-8 interacts with its cognate receptors CXCR1 and CXCR2 to promote tumor cell survival and induce epithelial–mesenchymal transition (EMT) [33,34]. Several signaling cascades are strongly implicated in EMT regulation, including the TGFβ–Syk/Src–AKT/ERK, p38/JNK–ATF-2, PI3K/AKT, and NF-κB pathways. Transcription factors such as hypoxia-inducible factor-1α (HIF-1α), nuclear factor κB (NF-κB), activator protein-1 (AP-1), signal transducer and activator of transcription (STAT), and β-catenin, all interconnected with these pathways, regulate gene expression controlling cell survival, angiogenesis, and proliferation [35]. Considering the key role of hypoxia-inducible factors (HIFs) in the reprogramming of cell metabolism and inflammatory responses, verification of intracellular HIF-1α stabilization is essential for confirming the biological validity of the hypoxia model. Under normoxic conditions, HIF-1α undergoes rapid ubiquitination and degradation via the prolyl hydroxylase- and von Hippel–Lindau (VHL)-dependent proteasomal pathway, while reduced oxygen availability inhibits this process, leading to HIF-1α accumulation, nuclear translocation, heterodimerization with HIF-1β, and activation of hypoxia-responsive gene transcription. It is noteworthy that hypoxia-induced stabilization of HIF-1α activates the expression of many genes essential for tumor adaptation, including vascular endothelial growth factor (VEGF), a key mediator of angiogenesis, lactate dehydrogenase A (LDH-A), erythropoietin (EPO), glycolytic enzymes, and glucose transporters GLUT1 and GLUT3, thereby promoting metabolic reprogramming. In addition, HIF-1α regulates the expression of nitric oxide synthase and insulin-like growth factor 2 (IGF2), which plays a key role in the proliferation and survival of cancer cells [36]. It is know that HIF-1α interacts with the NF-κB and STAT3 pathways, jointly regulating the expression of VEGF, IL-8, MMP-2, and other mediators, which our study significantly influenced. Thus, the oxygen-dependent changes in cytokine and MMP secretion observed here are consistent with the activation of these signaling networks that respond to hypoxia [37,38,39,40]. While CAPE is a potent NF-κB inhibitor under normoxia, our data suggest that in a hypoxic environment, the accumulation of HIF-1α may functionally override this inhibition, sustaining the pro-inflammatory drive despite CAPE treatment [40]. Consequently, the oxygen-dependent cytokine profiles observed here serve as a phenotypic readout of these underlying signaling networks. While we physically controlled hypoxia (1% O_2_), we acknowledge that direct verification of intracellular pathway activation (e.g., via Western blot for p-NF-κB/HIF-1α) remains a necessary next step to confirm this specific molecular interference. During EMT, epithelial cancer cells acquire mesenchymal traits that enable motility, invasion of surrounding tissues, and metastasis to distant organs. In this context, IL-8 acts as a key mediator facilitating tumor cell migration, invasion, and angiogenesis, ultimately enhancing metastatic potential. Recent studies have therefore identified CXCL8 as a promising molecular target for optimizing both conventional and immunotherapeutic strategies.

In the present study, CAPE at a concentration of 50 μg/mL (175.86 μM) followed by IFN-α stimulation under normoxic conditions significantly increased IL-8 secretion compared with the IFN-α control (*p* = 0.006). This observation may reflect CAPE-related modulation of immune activation pathways. However, it is well established that IL-8 expression correlates with enhanced angiogenesis, tumorigenicity, and metastatic potential in various cancers [34]. Importantly, hypoxia within the tumor microenvironment is closely associated with therapeutic resistance and aggressive tumor progression. Under reduced oxygen tension, cancer cells undergo metabolic reprogramming, shifting toward aerobic glycolysis and de novo lipid biosynthesis to maintain energy balance and redox homeostasis [7]. This metabolic shift promotes angiogenesis and erythropoiesis, enabling tumor adaptation and survival. Nevertheless, in our hypoxic model, CAPE did not induce statistically significant changes in IL-8 levels, suggesting that its modulatory capacity may be constrained under oxygen deprivation. Interleukin-26 (IL-26) is a pro-inflammatory cytokine and a recognized member of the IL-10 cytokine family, predominantly secreted by Th17 cells [41]. Elevated IL-26 expression has been reported in triple-negative breast cancer (TNBC) compared with normal tissue and peripheral blood [42]. In the current study, under normoxic conditions, CAPE—irrespective of concentration—significantly reduced IL-26 levels following either IFN-α or LPS stimulation. This is a biologically relevant observation, as IL-26 is known to induce inflammation, promote tumor growth, and enhance angiogenesis. In terms of its mechanism of action, IL-26 stimulates IL-22-producing cells, which subsequently suppress cytotoxic T-cell function and facilitate tumor progression through activation of anti-apoptotic signaling pathways [43]. Multivariate PCA further confirmed that IL-26 suppression represented the strongest and most consistent anti-inflammatory effect of CAPE, defining the second principal component (PC2) (Figure 4). This finding indicates that CAPE selectively attenuates IL-26-driven inflammatory signaling, underscoring its potential as a modulator of cytokine-dependent pathways in glioma biology.

The modulation of both pro-inflammatory and anti-inflammatory cytokines, including interleukin-10 (IL-10), has been strongly associated with patient outcomes and antitumor efficacy. IL-10 acts as a key intercellular regulator of immune and inflammatory responses, exerting context-dependent effects on tumor immunity [44]. Notably, both mice and humans deficient in IL-10 signaling develop spontaneous tumors with high frequency, emphasizing its protective, immunoregulatory role [45]. In the present study, CAPE under normoxic conditions significantly increased IL-10 levels following both IFN-α and LPS + IFN-α stimulation. However, this pattern was not reproduced under hypoxia, suggesting that oxygen availability critically determines CAPE’s immunomodulatory capacity. Human glioblastoma cell lines are commonly employed in vitro and in vivo to model mechanisms of tumor migration and invasion. In this context, cytokine signaling and matrix metalloproteinases (MMPs) are key molecular drivers of glioma progression. MMPs mediate degradation of extracellular matrix (ECM) components and disruption of the blood–brain barrier (BBB), facilitating tumor expansion and therapeutic resistance. Hypoxia has been shown to upregulate MMP-2 mRNA expression in U87, U251, U373, and LN18 glioblastoma cell lines through HIF-1α activation, thereby increasing invasive potential [46]. Expression of several MMPs, including MMP-1, -2, -7, and -9, correlates with tumor grade, whereas MMP-3 appears to play a limited role in glioblastoma progression. Our findings demonstrate that CAPE, particularly under IFN-α or combined LPS + IFN-α stimulation, enhances MMP-2 secretion, indicating that CAPE may influence glioma invasiveness through modulation of ECM-remodeling enzymes. Interestingly, Markiewicz-Żukowska et al. reported that ethanolic extracts of propolis combined with Hypericum perforatum significantly reduced the invasiveness of U87MG glioma cells by inhibiting MMP-2 and MMP-9 secretion and suppressing migration [47]. This suggests a possible synergistic effect of multiple propolis-derived constituents, rather than CAPE alone. In our previous studies, we demonstrated that both brown Polish and green Brazilian propolis exert beneficial effects on the astrocytoma microenvironment and may serve as potential immunomodulatory agents in the therapeutic strategy for gliomas of varying malignancy grades [48,49]. Collectively, these findings indicate that while CAPE can modulate cytokine and MMP profiles, its biological impact within the tumor microenvironment is context- and oxygen-dependent, and potentially enhanced in combination with other bioactive components of propolis.

In the present study, we evaluated the effect of CAPE on pentraxin-3 (PTX3) concentrations, given its recognized role as a biomarker of inflammation [50]. Pentraxins constitute a family of evolutionarily conserved proteins sharing a common structural motif, and PTX3 belongs to the long pentraxin subfamily. It plays a critical role in innate immunity and the regulation of inflammatory processes. PTX3 is released locally at sites of inflammation by immune, stromal, endothelial, and neoplastic cells. Elevated PTX3 levels have been associated with tumor progression and aggressiveness, reflecting its dual role in cancer biology [51,52]. Recent studies emphasize the involvement of PTX3 in angiogenesis and inflammation, highlighting its participation in the crosstalk between tumor and immune cells. Intriguingly, PTX3 can also act as an extrinsic tumor suppressor, counteracting macrophage-mediated, complement-dependent tumor-promoting inflammation [53]. PTX3 expression has been reported in numerous malignancies, including glioma, lung, ovarian, pancreatic, and prostate cancers, as well as myxoid liposarcoma and esophageal squamous cell carcinoma [54]. A wide range of inflammatory mediators—including IL-1β, TNF-α, toll-like receptor agonists, HDL, IL-10, and LPS—can induce PTX3 expression. Several clinical studies have demonstrated that PTX3 expression varies among glioma grades and correlates with histopathological classification and disease severity. Notably, glioblastomas (GBMs) display markedly higher PTX3 levels than low-grade astrocytomas or oligodendrogliomas [55]. In our experimental model, CAPE showed no statistically significant effect on PTX3 secretion at any tested concentration or stimulation condition when compared with respective control groups. These findings indicate that, under basal conditions (without pro-inflammatory stimulation), CAPE does not modulate PTX3 levels, consistent with its lack of effect on other measured mediators, including IL-8, IL-10, IL-26, MMP-1, MMP-2, and MMP-3.

Multivariate analyses (PCA and HCA) provided consistent and convergent evidence that oxygen availability represents a critical determinant of CAPE’s bioactivity profile. The results clearly demonstrated that CAPE exerts complex and context-dependent modulatory effects, enhancing MMP-1 (following IFN-α stimulation, *p* < 0.05), MMP-2 (after LPS + IFN-α co-stimulation, *p* < 0.001), and IL-10 (*p* < 0.05). Under normoxic conditions, the cellular response system appeared highly structured, characterized by two orthogonal and functionally distinct axes: a pro-inflammatory, LPS-driven pathway involving IL-8, MMPs, IL-10, and PTX3 (PC1), and a CAPE-mediated anti-inflammatory pathway associated with IL-26 suppression (PC2). By contrast, hypoxia induced a profound reconfiguration of the response network, shifting the equilibrium toward a pro-inflammatory phenotype. Under low oxygen tension, CAPE no longer suppressed cytokine activity but rather enhanced the secretion of key pro-inflammatory mediators, including IL-8, IL-26, MMP-2, and PTX3 (all *p* < 0.05). Its activity became polarized—potentiating pro-inflammatory mediators while suppressing IL-10 (with LPS, *p* < 0.01) and MMP-1 (25 µg/mL with LPS, *p* = 0.015). PCA revealed a fragmented and polarized network structure with two distinct inflammatory axes emerging: one IFN-α-driven, dominated by MMP-1, and another centered on the IL-8/MMP-3 cluster. HCA quantitatively confirmed this functional restructuring. The comparison between normoxic and hypoxic models thus provides coherent, multi-level evidence that oxygen availability acts as a molecular switch determining the immunomodulatory character of CAPE. Under normoxia, CAPE behaves predominantly as an anti-inflammatory modulator through suppression of IL-26, whereas under hypoxia its profile reverses toward a pro-inflammatory and pro-remodeling phenotype, amplifying IL-8 and MMP-2 expression. Hypoxia simultaneously reorganizes the entire response network, fragmenting and polarizing cytokine–MMP interactions. These findings highlight the necessity of considering microenvironmental parameters, such as oxygenation, when evaluating the pharmacological activity of natural compounds, particularly polyphenols. Environmental factors can fundamentally—and even oppositely—alter their biological effects. Limitation and future perspective: Although the present study provides important insights, it is limited by the use of a single cell line (CCF-STTG1). However, we would like to emphasize that the presented results should be treated as preliminary and serve to formulate hypotheses that require further verification in multiple glioma models in future studies. Moreover, we would like to mention the limitations of the cellular model, which include the lack of typical tissue histology, the lack of functional vascularization and cells mediating the adaptive immune response, as well as the lack of endocrine, paracrine, and nervous signaling and gradients of various substances found in a living organism. Therefore, extrapolation to in vivo conditions requires cautious interpretation. Furthermore, an additional limitation of the present study is the lack of direct assessment of CAPE stability, intracellular accumulation, and metabolic conversion under normoxic versus hypoxic conditions, which may influence compound availability and thus contribute to the observed oxygen-dependent effects.

## 4. Materials and Methods

### 4.1. Materials

Caffeic acid phenethyl ester (CAPE) was purchased from Sigma-Aldrich (Munich, Germany). DMSO was purchased from Sigma Chemical Company (St. Louis, MO, USA). Lipopolysaccharide (LPS; *E. coli* O26:B6) was obtained from Difco Laboratories (Detroit, MI, USA). Interferon-alpha (IFN-α; 3 MIU/0.5 mL) was purchased from Schering-Plough (Brinny, Ireland). The human astrocytoma cell line CCF-STTG1 was obtained from the American Type Culture Collection (ATCC, Manassas, VA, USA). Cells were cultured in RPMI 1640 medium supplemented with 10% fetal bovine serum (FBS) (ATCC, Manassas, VA, USA), 100 U/mL penicillin, and 100 μg/mL streptomycin (PAA Laboratories, Pasching, Austria). MTT was delivered from Sigma-Aldrich (St. Louis, MO, USA).

### 4.2. CCF-STTG1 Culture Collection

CCF-STTG1 cells, derived from a grade IV astrocytoma of a female patient, were cultured at 37 °C in a humidified atmosphere containing 5% CO_2_. Cells were maintained in monolayer cultures using RPMI 1640 medium supplemented with 10% FBS, 100 U/mL penicillin, and 100 μg/mL streptomycin. Subculturing was performed twice weekly. Cell counts were determined using a Bürker counting chamber according to Equation (1):

Number of cells in 1 mL = 4 squares counted 2 × 100 × 1000(1)

Suspensions containing 5 × 10^4^ cells/mL were prepared for the experiments. Cell density was adjusted to 1 × 10^5^ cells/mL, and 200 μL of the suspension was seeded into 96-well plates. Cells were stimulated with LPS (200 μg/mL) and/or IFN-α (100 U/mL) for 2 h, followed by treatment with CAPE (25 or 50 μg/mL) for 24 h. Moreover, to aim obtained hypoxic condition, after adding the compounds, the plate was placed in a hypoxic incubator with 1% oxygen for 2 h. After the hypoxic exposure, the plate was returned to standard culture conditions (5% CO_2_ and 95% air) for the remaining 22 h.

### 4.3. The Cytotoxicity Assay–MTT Assay

Cell viability was evaluated using the MTT assay, which measures the reduction of 3-(4,5-dimethylthiazol-2-yl)-2,5-diphenyltetrazolium bromide (MTT) to insoluble formazan crystals by mitochondrial dehydrogenases in viable cells. CAPE at concentrations of 25 μg/mL and 50 μg/mL, with or without LPS and/or IFN-α, was added to 96-well plates containing 200 μL of culture medium. Four control groups were included: vehicle control (DMSO), LPS control, IFN-α control, and LPS + IFN-α control. After 24 h, the medium was removed and replaced with 180 μL of fresh medium and 20 μL of MTT solution (5 mg/mL in PBS). Plates were incubated for 4 h, after which the supernatant was discarded, and DMSO was added to dissolve formazan crystals. Absorbance was measured spectrophotometrically at 550 nm, and cell viability was calculated according to Equation (2):

% cell viability = sample absorbance 100/absorbance of the control,(2)

### 4.4. Multiplex Bead-Based Cytokine Assay

Cytokine concentrations in the cell culture supernatant were determined using the Bio-Plex Human Cytokine Panel (Bio-Rad Laboratories, Hercules, CA, USA) in conjunction with the Bio-Plex 3D Suspension Array System, based on xMAP^®^ technology. Briefly, nine calibration standards and the magnetic bead suspension were prepared according to the manufacturer’s protocol. Then, 50 μL of standards and beads were added to each well of a 96-well plate and incubated for 2 h on a shaker. After washing with 100 μL Bio-Plex Wash Buffer using an ELx50 magnetic washer (Winooski, VT, USA), 50 μL of biotinylated antibody solution was added to each well and incubated for 30 min. Following three additional washes, 50 μL of streptavidin–phycoerythrin conjugate was added, followed by a final wash and resuspension of beads in 110 μL assay buffer. Fluorescence detection was performed using a two-laser system: the first laser (638 nm) identified bead regions (color-coded per analyte), while the second laser (532 nm) excited phycoerythrin to quantify fluorescence intensity proportional to the analyte concentration. Cytokine levels were calculated from standard calibration curves. All experiments were performed in three independent biological replicates (*n* =3), with each sample measured in technical triplicates.

### 4.5. Statistical Analysis

Experimental data obtained under normoxic and hypoxic conditions were analyzed as two independent datasets. The raw concentration values were used for the one-way analysis of variance (ANOVA), followed by Fisher’s least significant difference (LSD) post hoc test, to assess the statistical significance of differences between experimental groups. For multivariate analyses (PCA and HCA), data preprocessing was applied to ensure appropriate normalization and comparability between variables. Specifically, to ensure normal distribution approximation and homoscedasticity, the raw concentration values were first log-transformed (log_10_). Subsequently, to render variables with different magnitude ranges comparable, the data were standardized using Z-score normalization (assigning each variable a mean of 0 and a standard deviation of 1) prior to PCA and HCA algorithms. Principal component analysis (PCA) was performed to explore the structure of the dataset and identify variables contributing most strongly to sample differentiation. Hierarchical cluster analysis (HCA) was employed to visualize relationships among the analyzed biomarkers, using Ward’s agglomeration method and the squared Euclidean distance as the similarity metric. A *p*-value < 0.05 was considered statistically significant. All statistical analyses were carried out using Statistica 13.3 software (StatSoft Inc., Tulsa, OK, USA).

## 5. Conclusions

Oxygen availability appears to be a key factor modulating the bioactivity profile of caffeic acid phenethyl ester (CAPE); however, interpretation of the current results must take into account the limitations of the experimental model used. Under normoxic conditions, CAPE exhibits mainly anti-inflammatory properties, while hypoxia causes a marked shift towards a more complex and partially pro-inflammatory phenotype, which is reflected in increased secretion of selected cytokines (IL-8, IL-26) and matrix metalloproteinases (MMP-2). It should be emphasized that these observations are based on a single glioblastoma cell line (CCF-STTG1), which, given the marked heterogeneity of glioblastomas, limits the possibility of generalizing the results. Therefore, the presented data should be treated as preliminary and primarily serve to formulate a hypothesis, requiring further validation in a broader panel of astrocytoma and glioblastoma models. Furthermore, the in vitro cell model does not fully reflect the complexity of the tumor microenvironment in vivo, including the natural tissue architecture, functional vascularization, the presence of cells mediating adaptive immune responses, or dynamic endocrine, paracrine, and neuronal signaling, as well as physiological gradients of oxygen. These inherent limitations require careful interpretation of the results when applied to in vivo conditions. Despite these limitations, the study provides important observation suggesting that hypoxia may fundamentally reprogram the immunomodulatory potential of CAPE.

## Figures and Tables

**Table 1:** Mean concentrations [pg/mL], standard deviations (SD) of selected cytokines: IL-8, IL-10, IL-26, metalloproteinases MMP-1, -2, -3 and PTX3 by native and stimulated astrocytes cell line CCF-STTG1 by LPS or/and IFN-α in normoxic and hypoxic conditions corresponding to Figure 3.

Sample	IL-8		IL-10		IL-26		MMP-1		MMP-2		MMP-3		Pentraxin-3	
	Mean	SD	Mean	SD	Mean	SD	Mean	SD	Mean	SD	Mean	SD	Mean	SD
CAPE 25 µg/mL	2761.0	191.2	3.4	1.8	237.2	49.8	15.6	2.8	49.7	10.6	652.7	113.7	1042.6	695.7
CAPE 25 µg/mL + IFN-α	4066.9	814.6	6.9	5.2	118.6	60.8	65.2	26.0	150.1	7.0	937.5	307.9	1631.7	165.2
CAPE 25 µg/mL + LPS	4129.4	387.8	6.1	1.7	168.1	103.7	37.1	32.3	536.4	348.1	979.6	416.9	2226.9	132.7
CAPE 25 µg/mL + LPS + IFN-α	5106.6	151.6	12.0	1.6	217.0	102.3	91.9	14.7	2015.5	319.1	2081.6	451.7	2200.1	152.4
CAPE 50 µg/mL	3451.1	470.6	6.1	1.7	142.1	92.2	48.8	22.1	144.9	18.8	812.3	352.4	1402.5	270.5
CAPE 50 µg/mL + IFN-α	5038.8	2895.1	8.4	2.7	149.3	126.4	86.6	32.9	356.7	246.0	1251.3	659.1	1505.8	279.5
CAPE 50 µg/mL + LPS	4799.3	206.8	8.5	1.5	131.6	74.6	54.7	38.6	339.0	339.4	1273.7	541.3	1927.4	593.0
CAPE 50 µg/mL + LPS + IFN-α	4300.3	301.6	9.5	4.4	185.6	75.4	91.9	14.7	1911.2	546.2	1297.6	231.9	1912.3	1219.8
Control DMSO	2062.8	38.2	5.5	0.6	204.5	20.3	16.3	2.1	278.2	307.6	594.8	339.9	1364.2	39.8
Control IFN-α	2824.8	103.7	5.6	0.9	363.3	62.6	18.1	0.6	459.5	483.8	855.2	270.1	1364.2	132.1
Control LPS	4327.4	333.0	3.4	0.9	310.3	16.7	53.4	19.6	681.5	658.4	1146.4	144.3	2437.3	194.1
Control LPS + IFN-α	5516.6	231.6	6.8	3.0	285.1	99.0	68.7	48.7	696.4	552.4	1281.7	474.0	2525.9	330.5
CAPE 25 µg/mL hypoxia	2485.3	203.9	8.1	2.9	307.2	162.2	99.3	23.9	1308.9	616.6	694.6	60.3	1444.5	185.2
CAPE 25 µg/mL + LPS hypoxia	4196.4	238.1	8.0	2.3	225.5	37.5	44.5	27.7	1771.0	520.2	1566.5	94.6	2073.5	148.3
CAPE 25 µg/mL + IFN-α hypoxia	3464.6	342.3	11.7	2.6	60.3	3.6	77.2	30.1	1354.1	349.5	1125.8	241.1	1261.6	242.7
CAPE 25 µg/mL + LPS + IFN-α hypoxia	5008.3	290.5	13.1	1.1	274.5	134.6	91.9	14.7	1940.6	444.0	1782.9	65.6	2210.8	167.1
CAPE 50 µg/mL hypoxia	2532.6	305.7	5.9	0.9	95.9	45.1	70.7	31.9	1325.6	394.4	974.3	497.8	1476.3	94.5
CAPE 50 µg/mL + LPS hypoxia	3486.9	382.9	8.8	1.8	187.5	166.1	83.4	14.7	1003.6	260.0	1227.6	218.0	2667.1	812.3
CAPE 50 µg/mL + IFN-α hypoxia	4681.3	134.0	9.7	0.7	287.7	32.6	67.8	51.4	1950.7	120.1	1831.6	460.4	1399.9	167.1
CAPE 50 µg/mL + LPS + IFN-α hypoxia	4682.3	62.2	9.2	1.9	178.6	21.8	95.2	24.6	1353.5	701.5	1707.9	543.4	1491.5	858.7
Control DMSO hypoxia	2494.0	293.2	6.4	1.0	163.9	41.,0	88.7	41.0	1029.8	303.1	557.5	420.1	1393.9	68.1
Control IFN-α hypoxia	3418.3	295.7	12.5	1.1	100.5	37.2	88.7	41.0	707.,7	112.5	1237.8	355.,6	1588.3	45.4
Control LPS hypoxia	3813.0	249.1	13.5	3.9	191.2	21.8	110.4	28.7	1421.6	525.5	1711.7	748.7	2628.0	569.4
Control LPS + IFN-α hypoxia	4113.1	25.9	8.8	0.4	81.9	34.7	91.9	14.7	840.3	419.0	1202.4	229.1	1256.8	798.9

## Data Availability

Data are contained within the article.

## Abbreviations

The following abbreviations are used in this manuscript:

CAPE	Caffeic Acid Phenethyl Ester
GBM	glioblastoma multiforme
IL	interleukin
IFN	interferon
LPS	lipopolysaccharide
MMPs	metalloproteinases
PTX-3	pentraxin-3
NF-κB	nuclear factor kappa B
VEGF	vascular endothelial growth factor
TME	tumor microenvironment
COX-2	cyclooxygenase-2
BBB	blood–brain barrier
EMT	epithelial–mesenchymal transition
HIF-α	hypoxia-inducible factor-1α
TNBC	triple-negative breast cancer
